# Elusive Roles of the Different Ceramidases in Human Health, Pathophysiology, and Tissue Regeneration

**DOI:** 10.3390/cells9061379

**Published:** 2020-06-02

**Authors:** Carolina Duarte, Juliet Akkaoui, Chiaki Yamada, Anny Ho, Cungui Mao, Alexandru Movila

**Affiliations:** 1 Department of Periodontology, College of Dental Medicine, Nova Southeastern University, Fort Lauderdale, FL 33324, USA; ja1617@nova.edu (J.A.); cyamada@nova.edu (C.Y.); ah2197@nova.edu (A.H.); 2 Department of Medicine, The State University of New York at Stony Brook, Stony Brook, NY 11794, USA; Cungui.Mao@stonybrookmedicine.edu; 3 Cancer Center, The State University of New York at Stony Brook, Stony Brook, NY 11794, USA; 4 Institute for Neuro-Immune Medicine, Nova Southeastern University, Fort Lauderdale, FL 33324, USA

**Keywords:** ceramides, ceramidases, inflammation, neurodegenerative diseases, infectious diseases

## Abstract

Ceramide and sphingosine are important interconvertible sphingolipid metabolites which govern various signaling pathways related to different aspects of cell survival and senescence. The conversion of ceramide into sphingosine is mediated by ceramidases. Altogether, five human ceramidases—named acid ceramidase, neutral ceramidase, alkaline ceramidase 1, alkaline ceramidase 2, and alkaline ceramidase 3—have been identified as having maximal activities in acidic, neutral, and alkaline environments, respectively. All five ceramidases have received increased attention for their implications in various diseases, including cancer, Alzheimer’s disease, and Farber disease. Furthermore, the potential anti-inflammatory and anti-apoptotic effects of ceramidases in host cells exposed to pathogenic bacteria and viruses have also been demonstrated. While ceramidases have been a subject of study in recent decades, our knowledge of their pathophysiology remains limited. Thus, this review provides a critical evaluation and interpretive analysis of existing literature on the role of acid, neutral, and alkaline ceramidases in relation to human health and various diseases, including cancer, neurodegenerative diseases, and infectious diseases. In addition, the essential impact of ceramidases on tissue regeneration, as well as their usefulness in enzyme replacement therapy, is also discussed.

## 1. Introduction

**Figure 1:**
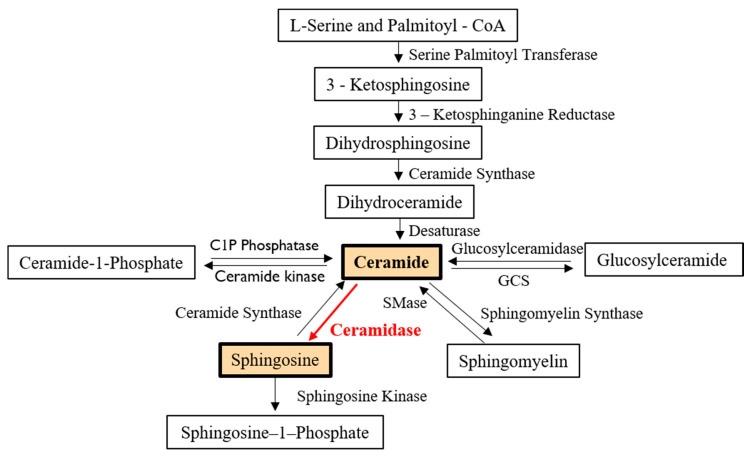
Role of ceramidases in ceramide metabolism. Ceramide in mammalian cells may be generated: (1) via the de novo synthesis pathway, which begins with the condensation of L-serine and Palmitoyl-CoA); (2) by the hydrolysis of sphingomyelin and glucosylceramide; or (3) from the dephosphorylation of ceramide-1-phosphate. Ceramidase is an enzyme that cleaves fatty acids from ceramide, producing sphingosine. Sphingosine may then be phosphorylated by a sphingosine kinase to form sphingosine-1-phosphate. SMase—sphingomyelinase; GCS—glucosylceramide synthase.

Ceramides are bioactive sphingolipids responsible for cell apoptosis, senescence, and autophagy [1]. They are the precursors of other bioactive sphingolipids, including sphingosine (SPH), sphingosine-1-phosphate (S1P), and ceramide-1-phosphate, which play specific roles in signal transduction pathways (Figure 1) [1]. 

The de novo anabolic pathway for the biosynthesis of ceramide begins with the condensation of the amino acid, L-serine, and palmitoyl-CoA, producing 3-ketosphingonine. Then, 3-ketosphingonine is quickly converted into dihydrosphingosine (dhSPH) by 3-ketosphinganine reductase. The subsequent acylation of dhSPH by (dihydro)ceramide synthases gives rise to dihydroceramides. Finally, the removal of two hydrogens from a fatty acid chain of the dihydroceramides by the enzyme desaturase results in the formation of ceramides (Figure 1) [2]. Ceramides can be further hydrolyzed into sphingosine (SPH) and free fatty acids by ceramidases (Figure 1). SPH is the most common sphingolipid base molecule in mammalian cells and is the precursor of S1P [1]. 

**Figure 2:**
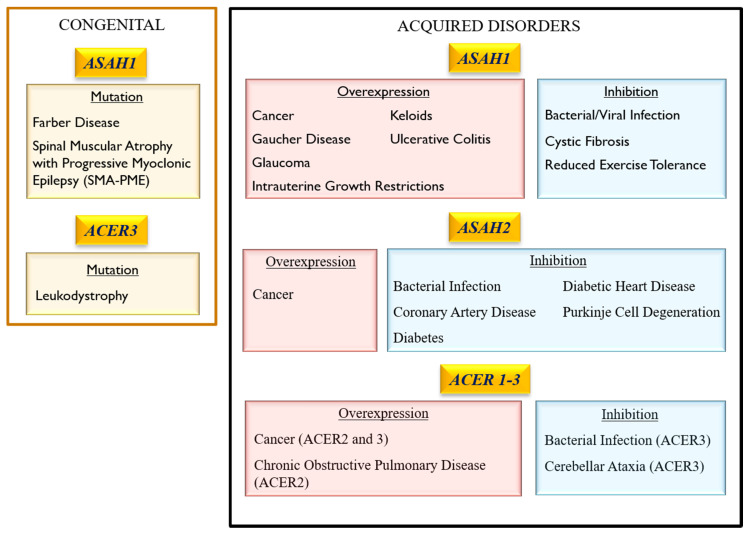
Pathological consequences of ceramidase dysregulation in mammalian cells which occur upon a loss or a gain of function.

The bioactive lipid mediator S1P is involved in cell proliferation, differentiation, and survival, whilst ceramides and SPH mediate cell death [1,2]. Notably, SPH is exclusively generated from the catabolism of ceramides by ceramidases [2]. Ceramidases control the balance between S1P and ceramides/SPH concentration, which leads to either cell survival or cell death [1,3]. Hence, a ceramidase-based enzyme replacement therapy that simultaneously achieves ceramide reduction and SPH elevation has been recently examined [4]. This therapeutic approach intends to reduce the negative pathophysiological impact of cell death mediated by ceramides [4]. To date, five human ceramidases have been identified and classified according to their optimal pH for catalytic activity: one acid ceramidase (ACDase) encoded by the gene ASAH1, one neutral ceramidase (NCDase) encoded by ASAH2, and three alkaline ceramidases (ALKCDase) encoded by the genes ACER1, ACER2 and ACER3 [3]. ACDase is the most widely studied ceramidase, and its effects on pathophysiology are variable. While NCDase and ALKCDases 1–3 have been the subject of studies in recent decades, our knowledge about their roles in pathophysiology remains limited. Thus, the aim of this review was to summarize the most recent knowledge of the biology of ceramidases and their role in the pathology of various common human diseases, including cancer, diabetes, and neurodegenerative diseases (Figure 2). In addition, the roles of the ceramidases in infectious diseases, tissue regeneration, and healing were also addressed. 

## 2. Characteristics and Regulatory Pathways of Ceramidases

### 2.1. General Characteristics

#### 2.1.1. Acid Ceramidase

ACDase (ASAH1) is synthesized from a 53–55 kDa polypeptide precursor which is proteolytically processed into the enzyme’s 13 kDa α-subunit and 30 kDa β-subunit inside the lysosomes [5,6]. ACDase is a lipid hydrolase found in the lysosomal compartment of cells which catalyzes the hydrolysis of C_6:0_–C_18:0_ ceramides to SPH [6]. It is ubiquitously expressed in human tissues, with a particularly high expression in the heart and kidneys and is known for its role in senescence and apoptosis [7]. 

#### 2.1.2. Neutral Ceramidase

NCDase (ASAH2) is synthesized as 118 kDa and 142 kDa isoforms in humans and as a 96 kDa molecule in mice [6]. NCDase is a transmembrane glycoprotein that is highly expressed in the human intestinal system and uses various ceramides and dihydroceramides as a substrate, with a reported preference for C_16:0_ and C_18:0_ ceramides [8,9]. It is localized to different cellular compartments, including the plasma membrane of cells; regulates the conversion of ceramide into SPH and S1P; and is important for the metabolism of dietary sphingolipids [10]. 

#### 2.1.3. Alkaline Ceramidase

ALKCDases 1–3 (*ACER*1, ACER2, ACER3) are the smallest proteins among the ceramidases, with molecular weights of 31–31.6 kDa [6,11]. ALKCDases are predominantly located in the Golgi complex and endoplasmic reticulum and play a role in cell differentiation [11]. ACER1 hydrolyses C_20:0_-C_24:0_ ceramides [9]. It is predominantly expressed by skin cells and is involved in their differentiation, as well as in the viability of hair follicle stem cells [6,12,13,14]. ACER2 hydrolyses ceramides and dihydroceramides C_18:1_ and C_20:1_ [9]. It is upregulated during DNA damage and induces programmed cell death through an SPH-dependent pathway [15]. ACER3 hydrolyses ceramides, dihydroceramides, and phytoceramides with long unsaturated acyl chains [9]. It has been described as a seven-transmembrane protein, much like the adipocyte receptor (ADIPOR), and is associated with cytokine upregulation [16].

This brief overview of the general characteristics of ceramidases indicates that they have been classified according to their optimal pH. However, ceramidases also differ in molecular weight and expression patterns. Importantly, all three groups of ceramidases have a specific ceramide affinity and reported cellular functions. It is important to highlight that the ALKCDases, although classified together, differ in ceramide affinity and function. Moreover, ALKCDase-3, like NCDase, is a transmembrane protein and not a soluble enzyme. Thus, further consideration should be given to the classification of ceramidases and, particularly, the ALKCDases. 

### 2.2. Regulatory Pathways

#### 2.2.1. Acid Ceramidase

The activation of ACDase induces a pro-survival state, while its inhibition leads to cell death through a variety of apoptotic pathways mediated by caspases (CASP), poly (adp-ribose) polymerase (PARP), or cathepsins (CTS) [10,17,18,19,20,21,22,23,24,25]. Cathepsin B and Cathepsin D are activated during ceramide-induced apoptosis but are inhibited by ACDase activity [19,25]. Interestingly, the downregulation of Cathepsin B by ACDase increases ACDase’s own activation, triggering a feedback mechanism through which ACDase prolongs its own activation through Cathepsin B inhibition [10]. Additionally, ACDase activity can be regulated by Ceramide Synthase 6 (CerS6) [26]. CerS6 increases the levels of C_16:0_, which, in turn, activate ACDase through JNK-AP1-dependent mechanisms. However, this same mechanism mediates the inhibition of the gene expression of NCDase and ALKCDases in colorectal adenocarcinoma [26]. 

An age-dependent inhibition of ACDase leads to ceramide accumulation, an increase in oxidative stress, and the death of retinal cells and erythrocytes [27,28]. By contrast, it was reported that kidney cells collected from aged mice show an elevated expression of *Asah1* mRNA compared to that of young mice [29]. Thus, this published evidence suggests a tissue-specific ACDase activity in relation to cellular senescence and aging. 

#### 2.2.2. Neutral Ceramidase

The activity and gene expression of NCDase have been linked to cell-cycle arrest and growth regulation [30]. Biochemically, NCDase is a lipid amidase with a mechanistic similarity to a bacterial NCDase [8]. NCDase activates nitric oxide (NO), the WNT/β-catenin pathway, caspase apoptotic pathways, and autophagosomal activity in vivo and is associated with mitochondrial integrity [31,32,33,34]. Its gene expression and activity are regulated by c-Jun/AP-1 signaling, NO, all-trans retinoic acid, and ultra-violet radiation [35,36]. 

#### 2.2.3. Alkaline Ceramidase

The ALKCDases 1–3 are regulated through markedly different mechanisms. ACER 1 is upregulated by extracellular calcium, through which it contributes to the regulation of cell differentiation and growth arrest [37]. Meanwhile, ACER2 is induced by p53 and activates p38 MAPK and AP-1 signaling to mediate DNA damage response, autophagy, and apoptosis [15,38,39]. ACER3 is associated with the AKT/BAX pathway and activates the S1P phosphorylation of AKT through S1PR2 and PI3K in cancer cells [40,41]. 

## 3. Association of Ceramidase Gene Mutations with Human Inheritable Diseases

### 3.1. Acid Ceramidase

#### 3.1.1. Farber Lipogranulomatosis (FRBRL)

FRBRL is an autosomal recessive lysosomal disorder with a broad spectrum of phenotypes caused by 16 identified mutations of *Asah1* [5]. It is characterized by a substantial neurologic deficit, subcutaneous nodules, progressive arthritis with joint deformities, laryngeal hoarseness, and an accumulation of storage-laden CD68^+^ macroglia/macrophages in white matter, periventricular zones, and meninges of the brain [42]. Animal models of ACDase deletion present hematopoietic organ hypertrophy, characterized by a foamy macrophage infiltration and increased myeloid progenitor colonies [43]. These myeloid progenitor colonies are comprised of cells that can develop normally when treated with ACDase [43]. Additionally, ACDase deletion causes an impaired airway resistance, elastance, and compliance; reduced blood oxygenation; lung edema; and increased immune cell infiltration of the lungs by foamy macrophages and neutrophils [44]. Furthermore, an increased vascular permeability of the lungs, heart, thymus, liver and spleen, as well as neurologic problems, including decreased deambulation, anxiety, and impaired motor coordination, are also observed [42]. These neurologic problems are caused by abnormal sphingolipid profiles in the brain and CD68+ microglia [42]. 

Changes in the *Asah1* gene expression in FRBRL patients result in the upregulation of the inflammatory cytokines interleukin 4 (IL-4), IL-6, tumor necrosis factor alpha (TNFα), and macrophage colony stimulating factor (M-CSF) in addition to the angiogenic marker, vascular endothelial growth factor (VEGF) [42,45]. Likewise, the expressions of the chemo-attractants, monocyte chemotactic protein-1 (MCP-1), and interferon gamma-induced protein 10 (IP10), are inversely correlated with the level of ACDase activity [45]. These mediators of inflammation, angiogenesis, and insulin resistance may be associated with the immune cell infiltration found in the organ tissues of FRBRL animal models [45]. MCP-1 deletion can partially rescue FRBRL phenotypes by improving organomegaly, blood cell counts, and liver and lung damage by inflammatory infiltrates, as well as the behavioral and neurologic aspects of the disease. However, hematopoiesis is not improved [46]. Similarly, the overexpression of MCP-1, IP-10, and IL-6 can be partially corrected by hematopoietic stem cell transplants [45]. Moreover, treatment with ACDase induces a dose-dependent decrease in hematopoietic organ weight, macrophage infiltration, and *MCP-1* expression, as well as increased expression of Collagen Type 2 *(Col2),* aggrecan, and Sox-9 by chondrocytes [4]. 

#### 3.1.2. Spinal Muscular Atrophy with Progressive Myoclonic Epilepsy (SMA-PME) 

SMA-PME is a rare autosomal recessive disorder that is frequently associated with FRBRL and is caused by two identified mutations of *Asah1* [5]. This disorder is characterized by motor neuron disease and progressive myoclonic epilepsy, with a variable occurrence of sensorineural hearing loss, action tremor, cognitive dysfunction, and cerebral/cerebellar atrophy. Patients with SMA-PME present a 70–95% reduction in ACDase activity, a low ACDase/β-galactosidase ratio, and increased creatine kinase levels [47]. Additionally, the muscle atrophy associated with SMA can be accompanied by cyclooxygenase deficiency [48].

#### 3.1.3. Intrauterine Growth Restrictions (IUGR)

The consequence of ACDase gene overexpression during gestation and its therapeutic effect on associated genetic disorders has also been described. IUGR can result from the TGFβ/ALK5-mediated overexpression of *Asah1* mRNA and increased ACDase activity, which upregulates SPH but not S1P concentrations during pregnancy [49]. S1P is not upregulated at the same rate as SPH in IUGR due to the inactivation of SPH kinase 1 through the ALK1-SMAD1/5 pathway [49]. 

This suggests that ACDase may induce embryonic cell death through SPH rather than affect embryonic cell proliferation and differentiation through S1P in IUGR.

#### 3.1.4. Krabbe Disease 

Globoid cell leukodystrophy, or Krabbe disease, is a congenital disorder caused by mutations in the galactosylceramidase gene, *GALC*, and is characterized by psychomotor regression, muscular hypertonia, muscular spasticity, truncal hypotonia, irritability, seizures, and nystagmus [50]. This disorder is caused by an accumulation of psychosine, a by-product of the deacylation of GALC by ACDase [51]. Therefore, the inhibition of ACDase activity, as observed in FRBRL or after treatment with ACDase inhibitors, can rescue the Krabbe Disease phenotype by preventing psychosine accumulation [51]. 

### 3.2. Neutral ceramidase

There are no reports demonstrating the association of the point genetic mutations of ASAH2 with inheritable diseases in humans. It is important to mention that *Asah2^−/−^* mice are viable and appear without severe defects [52]. 

### 3.3. Alkaline Ceramidase

#### Progressive Leukodystrophy 

Progressive leukodystrophy is a group of disorders that affect the white matter of the brain and can occur as a consequence of ACER3 deficiency [53]. This condition is caused by a loss of function mutation in p.E33G which inactivates the catalytic activity of ACER3 and leads to an accumulation of sphingolipids in the blood [53]. The clinical phenotype associated with ACER3 mutations is caused by incorrect central nervous system myelination due to abnormal levels of ceramides in the brain [16]. While the study reporting the loss of function mutation in p.E33G did not report a sphingolipid accumulation or pattern in the brain, it is reasonable to assume that sphingolipid accumulation due to ACER3 inactivation results in abnormal sphingolipid patterns in the brain. 

## 4. Role of Ceramidase Activity in Human Non-heritable Diseases 

### 4.1. Role of Ceramidases in Cancer Pathology

**Figure 3:**
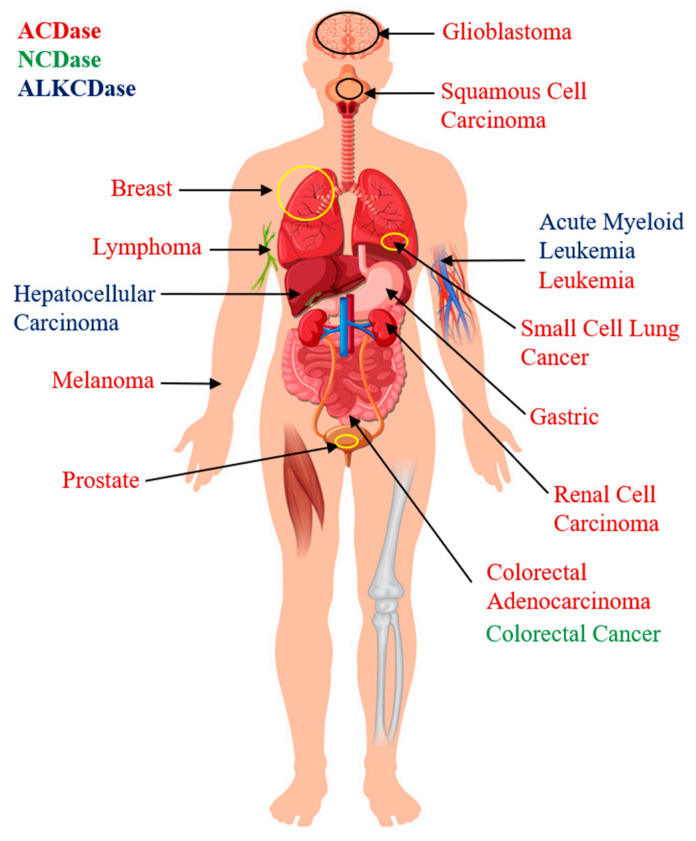
Overexpression of acid (ACDase), neutral (NCDase), and alkaline (ALKCDase) ceramidases in specific types of cancer.

The overexpression of ceramidases have been identified in various cancer cell types, and growing evidence suggests that they can be considered molecular markers and/or therapeutic targets for cancer [54] (Figure 3).

#### 4.1.1. Acid Ceramidase

ASAH1 has been identified in cancer cells and is associated with radiotherapy/chemotherapy-resistant tumors [22,55,56,57], metastatic cell lines [58], and estrogen/progesterone/androgen receptor-positive cells [59,60]. While ACDase gene overexpression has been identified in low-survival-rate colorectal adenocarcinoma and glioblastoma [57,61], it has also been observed in node-negative melanoma and breast cancer [59,62], which makes it a questionable marker for the aggressiveness or invasiveness of the disease. The *ASAH1* mRNA expression in cancer cells can be increased by radiotherapy, thereby generating resistance [56]. Likewise, the overexpression of *ASAH1* can be driven by the oncogene microphthalmia-associated transcription factor (MITF) [63]. ACDase activity is increased by the androgen receptor activation by dihydrotestosterone in prostate cancer, leading to decreased C_16:0_ levels and reduced cell apoptosis [60]. Incidentally, the ACDase activity is significantly more upregulated than the *ASAH1* expression in melanoma cells [62]. This may suggest that gene expression alone should not be the determining factor in the use of ACDase as a marker for cancer; ACDase activity should also be assessed.

Multiple molecular mechanisms by which ACDase activation regulates cancer development and progression have been identified. For instance, drug resistance in leukemia is mediated by the ACDase activation of the drug transporter molecule ATP-Binding Cassette, Subfamily B, Member 1 (ABCB1), through nuclear factor kappa B (NF-κB) [64], whilst leukemic cancer cell survival is increased by the ACDase-mediated upregulation of the myeloid cell leukemia sequence 1 (MCL-1) [10]. Furthermore, cancer cell necrosis is mediated by ACDase gene overexpression in polynuclear giant cancer cells that undergo asymmetric cell division [65]. ACDase also regulates cancer cell motility through the activation of the ITGαVβ5/FAK signaling cascade [63]. Additionally, the significant roles of ACDase in angiogenesis, chronic inflammation, and tumorigenesis may contribute to cancer development and progression [66,67]. ACDase affects multiple factors in cancer pathogenicity, which adds to the complexity of the enzyme in the diagnosis and treatment of the disease.

A variety of ACDase inhibitors have been developed and successfully tested in different cancer cell types. ACDase deletion blocks the cell cycle at G1/S, promotes senescence through the β-Galactosidase/MITF pathway, induces apoptosis, reduces tumorigenesis, increases growth arrest, and decreases malignancy [68]. It was demonstrated that the activity of ACDase was significantly inhibited by Carmofur [55,58], LCL521 [19,65,69,70], Ceranib2 [24,71,72,73], N-oleocylethanolamine (NOE) [22,57], ARN14988 [74], LCL204 [10,64], Monascus Purperus (MP) [18], Hesperetin (Hst) [17], Hesperetine-7-O-acetate (HTA) [17], Silibinin [20], Curcumin [23], and Sanguinarine [21,75], leading to an increased accumulation of intracellular ceramide and apoptosis in various types of cancer cells, including glioblastoma; squamous cell carcinoma; acute myeloid leukemia; colorectal adenocarcinoma; and breast, prostate, lung, gastric, and kidney cancer. Furthermore, Carmofur, NOE, LCL521, and Ceranib2 have been used in combination with chemotherapeutic drugs or photodynamic therapy to either overcome cancer cell resistance to treatment, increase cell sensitivity to specific drugs, or increase the overall effectiveness of cancer cell apoptosis [22,55,58,70,72,73]. Ceranib2 treatment leads to an abnormal cell and mitochondria morphology and decreases the ability of cells to cluster [24,74]. It activates PARP and CASP3/7/8/9-mediated cell apoptosis; increases the expression of the pro-apoptotic markers BID, BCL2-Associated Agonist Of Cell Death (*BAD*), and BCL2-Associated X Protein (BAX); and decreases the expression of anti-apoptotic protein B-Cell Cll/Lymphoma 2 (BCL-2) [72,73]. Furthermore, MP, Hst, HTA, Curcumin, and Sanguinarine activate the apoptotic pathways dependent on Casp3/9 or reactive oxygen species (ROS) [17,18,21,23,75]. Sanguinarine induces peroxide-dependent ceramide generation and the inhibition of the AKT activation pathway [75]. NOE and LCL204 induce PARP- and Casp3-mediated apoptosis [10,22], whereas LCL521 increases C_16:0_ levels, autophagosome accumulation, ER stress, and Cathepsin B- or Cathepsin D-mediated apoptosis [19]. Altogether, ACDase inhibitors are effective promoters of cancer cell death through different apoptotic pathways and have been shown to affect not only apoptosis but also cancer treatment resistance and cancer cell adhesion. 

#### 4.1.2. Neutral Ceramidase

An elevated gene expression of NCDase has been identified in both the plasma membrane and Golgi apparatus of colorectal cancer (CRC) cells, where its overexpression inhibits ceramide C6-mediated cell death [8]. Meanwhile, its deletion induces caspase and autophagosome-mediated apoptosis in the presence of C6 [32]. NCDase regulates CRC cell proliferation through the WNT/β-catenin pathway and by increasing the accumulation of SPH and S1P [31,32]. NCDase inhibition may affect cell-to-cell adhesion by reducing the β-catenin levels through AKT phosphorylation and, subsequently, GSK3β activation [31]. It also significantly reduces Azoxymethane-induced colon carcinogenesis by inhibiting aberrant crypt foci formation and transformation [32]. NCDase inhibition does not affect non-cancerous cell function, which makes it a suitable target for colon cancer therapy [32]. 

We can conclude that NCDase inhibition, like that of ACDase, activates apoptosis and affects adhesion in cancer cells. In addition, it may be a contributing factor in cancerous transformation.

#### 4.1.3. Alkaline Ceramidase

ALKCDases can also affect cancer development and treatment. ACER2 is upregulated by the tumor suppressor gene p53 [38,39]. It was demonstrated that a moderate upregulation of ACER2 increases the levels of SPH and S1P and inhibits cell cycle arrest and senescence. However, when overexpressed, ACER2 mediates programmed cell death, autophagy, and apoptosis through ROS [15,38,39]. ACER2 also contributes to the effects of ionizing radiation treatment [39]. It also increases the phosphorylation of Ezrin-radixin-moesin through intracellular S1P production, hereby inactivating this group of proteins that regulate cell shape and motility and have been associated with cancer progression and metastasis [76]. ACER3 is expressed in low-survival hepatocellular carcinomas and acute myeloid leukemia [40,41]. It induces the S1P phosphorylation of AKT through the S1P receptor 2 and PI3K and inhibits the AKT/BAX apoptotic pathway in cancer cells [40,41]. Therefore, the inhibition of ACER3 reduces cell growth and increases cancer cell apoptosis. 

These published observations indicate that ALKCDases are also associated with the regulation of cancer cell apoptosis. However, the observations of ACER2 overexpression reflect molecular effects contrary to those expected of ceramidases. Nonetheless, ALKCDases are associated with drug resistance and cancer metastasis, like the previously described ACDase.

### 4.2. Role of Ceramidases in the Onset of Age-Related Neurodegenerative Diseases 

Ceramidases are involved in myelin and fatty acid metabolism and are associated with changes in the brain during aging [77]. For instance, ACER3 is upregulated with age and leads to a decrease in the brain levels of C_18:0_ and C_18:1_ ceramide, and its deletion results in purkinje cell degeneration and impaired motor coordination and balance in mice [78]. It has been reported that the overexpression of ACDase has implications for the onset and progression of neurodegenerative diseases, including Alzheimer’s disease (AD) and Gaucher disease. Furthermore, treatment with ACDase inhibitors can control AD and Gaucher Disease, as well as Type IV Mucolipidosis.

#### Acid Ceramidase

AD is a multifactorial, highly heterogeneous, and complex disorder that affects the memory and cognitive functions of patients to the extent that they are completely dependent upon nursing care. It is now estimated that nearly 35.6 million patients are affected by AD worldwide and that about 4.6 million new cases are added each year, causing enormous societal and economic burdens, with the estimated cost reaching $1 trillion/year [79]. AD is caused by an accumulation of derivates from the amyloid precursor protein (APP), which can be modulated by the ATP-binding cassette transporter-2 (ABCA2). ABCA2 is a phospholipid transporter which increases the transcription of APP by activating the ACDase-mediated production of SPH [80]. Furthermore, ACDase inhibition by Ceranib 1 decreases SPH concentration and, subsequently, APP production in ABCA2-overexpressing cells [80].

Gaucher disease is a disorder caused by a loss of function mutations in the glucocerebrosidase (GCase)-encoding gene, Gba1. In a GCase deficiency, the breakdown of glucocylceramide (GlcCer) into ceramide and glucose by GCase is replaced by the ACDase deacylation of GlcCer into glucocylSPH (Glc-Sph), a cytotoxic compound [80]. The inhibition of ACDase by Carmofur corrects the lipid abnormalities in the GCase deficiency by reducing the accumulation of Glc-Sph [81,82]. GBA1 mutations are also a risk factor for Parkinson’s disease, a neurodegenerative disorder characterized by Lewy body inclusions containing α-synuclein. Treatment with ACDase inhibitors decreases the accumulation of α-synuclein in cases of GBA1 mutation [81]. Similar lipid patterns are observed in the optic nerves of glaucoma patients, where Asah1 and Asah2 genes are overexpressed, but non-lysosomal GCase-GBA2 is inhibited, resulting in a lower total lipid content and significantly higher concentrations of Glc-Sph [83]. 

Type IV Mucolipidosis is a neurodegenerative disease caused by a loss-of-function mutation of human transient receptor potential-mucolipin-1 (TRPML-1). Treatment with the ACDase inhibitor, carmofur, induces the activity of TRPML-1 tunnels by increasing the SPH concentration in kidney cells and acting as a mediator of lysosome fusion and trafficking in multivesicular bodies, which can potentially compensate for the loss of function of TRPML-1 [84].

### 4.3. Role of Ceramidases in Cardio-Pulmonary Disease

Elevated levels of ceramide are known to be correlated with adverse cardiac events, whereas SPH has been shown to increase intracellular NO levels and maintain the mitochondrial integrity of the cardiovascular system [33]. Conversely, increased blood S1P levels are associated with the pathogenesis of inflammatory and cardiovascular diseases [85]. Hence, an association between ceramidase and cardiopulmonary events is expected.

#### 4.3.1. Acid Ceramidase

The inhibition of ACDase activity is associated with cystic fibrosis (CF), which is caused by a dysregulation of the epithelial fluid transport in the lungs, resulting in a sticky dry mucous accumulation [86]. In CF, β1-Integrins are ectopically expressed in the luminal pole of epithelial cells and downregulate ACDase, leading to an increased ceramide accumulation. However, treatment with recombinant ACDase internalizes the β-Integrins and regulates ceramide accumulation, rescuing the CF phenotype [86]. 

#### 4.3.2. Neutral Ceramidase

NCDase is inhibited in coronary artery disease vessels. NCDase and ADIPOR mediate the NO-dependent flow-induced dilation (FID) through S1P. Meanwhile, NCDase inhibition induces the damaging peroxide-dependent FID [33]. In addition, the inhibition of NCDase also leads to mitochondrial dysfunction in diabetic hearts through a lactocylceramide accumulation [87]. 

#### 4.3.3. Alkaline Ceramidase

A high expression of ALKCDase genes, particularly ACER2, has been observed in cardiac tissue during hypoxia, where it plays a protective role [88]. However, an overexpression of ACER2 has been associated with chronic obstructive pulmonary disease (COPD) [89]. ACER2 inhibition contributes to a reduction in the circulating S1P and its analogue, dhS1P, as well as their precursors, SPH and dhSPH, in hematopoietic cells and reduces the concentration of dhS1P in the lungs [85,88]. 

These data indicate that ACDase and ALKCDase are increased in CF and COPD, respectively, whereas NCDase is decreased in coronary artery disease. 

### 4.4. Role of Ceramidases in Metabolic Disorders

#### 4.4.1. Acid Ceramidase

Multiple factors are involved in the onset and progression of metabolic disease, including the activities of ceramidases. Genetic variations of *ASAH1* have been associated with exercise tolerance and skeletal/cardiac muscle adaptation to exercise, which can condition adherence to physical activity regimens necessary for a healthy lifestyle, thereby increasing the individual risk of metabolic diseases [90]. After onset, metabolic disorders affect the physiology of the cardiovascular system, kidneys, and liver. Hyperglycemia inhibits the Unc51-Like Autophagy-Activating Kinase 1 (ULK1) phosphorylation in aortic endothelial cells, which leads to a dysregulation of autophagy and atherogenesis. However, ACDase activity can increase the phosphorylation of ULK1 and restore its function even in nutrient-rich conditions, thus preventing atherogenesis [91]. In addition, obesity-induced kidney damage is caused by hyperglycemic conditions that stimulate the NLR Family Pyrin Domain-Containing 3 (NLRP3) inflammasomes to release IL-1β in podocytes, but the treatment of podocytes with ACDase decreases the NLPR3-induced cytokine release through extracellular vesicles [92]. ACDase reduces the activity of Pannexin-1 (Panx1), a transmembrane channel glycoprotein that activates NLRP3 through S1P accumulation [93]. Animal models of ACDase deficiency show significant damage to the liver and change to lipid profiles and metabolism, including hepatomegaly with higher serum levels of aspartate, aminotransferase, alanine aminotransferase, and alkaline phosphatase and decreased levels of free fatty acids, triglycerides, and cholesterol [25]. The inducible liver-specific overexpression of ACDase in the Alb-AC transgenic mice, results in significantly reduced C_16:0_ ceramide in the liver and improved total body glucose homeostasis and insulin sensitivity under a high-fat diet [94]. However, aberrant ACDase overexpression in very low-density lipoprotein (VLDL) deficiency may result in non-alcoholic fatty liver disease, which can be normalized by supplementation with Vitamin E [95]. Adipocyte-specific ACDase overexpression improves glucose metabolism by white adipose tissue, reverses insulin resistance, reduces lipid accumulation in the liver, and reduces adipose inflammation and fibrosis [94]. This could be due to an ACDase-mediated activation of the adiponectin receptor that triggers an AMP-dependent kinase pathway, which subsequently inhibits adipogenesis and induces fatty acid oxidation [96]. 

#### 4.4.2. Neutral Ceramidase

Palmitate is a precursor of palmitoyl-CoA, a thioester used in the de novo biosynthesis of ceramide that is associated with pancreatic β-cell apoptosis and insulin resistance. Palmitate inhibits NCDase gene expression and activity in pancreatic β cells, which, in turn, exacerbates apoptosis through ceramide accumulation [97]. Pancreatic β cells secrete NCDase via exosomes that reduce palmitate-induced ROS and act as a protective mechanism against free fatty acid-induced apoptosis [98,99]. An overexpression of NCDase inhibits palmitate-induced apoptosis and may be a therapeutic target for type 2 diabetes mellitus and lipotoxicity [97]. Furthermore, Asah2 is one of the four genes related to sphingolipid metabolism that are deregulated in animal models of type 3 maturity-onset diabetes of the young [100]. This pathology is characterized by increased ceramide and SPH levels as well as hypochromic microcytic anemia, with abnormally-shaped and osmotically fragile red blood cells characterized by an accumulation of SPH [100]. 

#### 4.4.3. Alkaline Ceramidase

Non-alcoholic fatty liver disease is associated with an increased expression of ACER3, which reduces the accumulation of C_18:1_-ceramide in the liver [101]. Acer3 deletion reduces inflammation, fibrosis, oxidative stress, and apoptosis of hepatocytes through a palmitic acid-induced increase in C_18:1_-ceramide [101]. 

Altogether, we can conclude that the holistic beneficial effects of ACDase in metabolic disease have been demonstrated. ACDase activity controls atherogenesis, kidney damage, and liver damage, while improving glucose and lipid metabolism. In addition, NCDase also appears to improve metabolic conditions via a protective effect on pancreatic β cells, while ALKCDase3 mediates liver damage. 

## 5. Role of Ceramidase Activity in Infectious Diseases

### 5.1. Role of Ceramidase Activity in Bacterial Infection

#### 5.1.1. Acid Ceramidase

Ceramidases have been identified as contributors to bacterial infection and mediators of the immune response and inflammation. The α-toxin released by *Staphylococcus aureus* inhibits ACDase gene expression, causing decreased levels of SPH that contribute to bacterial infection susceptibility [102]. Moreover, this mechanism further increases the risk of infection by *S. aureus* and *Pseudomonas aeruginosa* in already ACDase- and SPH-deficient CF patients [86,102]. *Porphyromonas gingivalis*, an etiological factor for periodontitis, downregulates ACDase in periodontal tissues, thereby increasing its own apoptotic potential and inhibiting the host’s inflammatory response [103]. The inhibition of ACDase by bacteria increases host cell apoptosis and reduces the production of inflammatory cytokines, such as TNF-α, IL-1β, IL-6, and IL-17A, which delay the immune response [67,103]. Conversely, ACDase overexpression upregulates the inflammatory cytokines involved in the recruitment of neutrophils and macrophages, as demonstrated in ulcerative colitis, where ACDase mediates the associated histopathological characteristics of the disease [67]. 

#### 5.1.2. Neutral Ceramidase

Ceramide accumulation is increased after burn injuries and may be associated with bacterial infections that frequently lead to death. NCDase treatment protects against *Pseudomonas aeruginosa* infection after burn injuries by controlling ceramide accumulation and inducing the accumulation of SPH, which directly kills bacteria [104].

#### 5.1.3. Alkaline Ceramidase

Bacterial lipopolysaccharides may also downregulate the expression and activity of *Acer3* and increase C_18:1_ ceramide accumulation in mice [105]. A loss of *Acer3* expression leads to the production of pro-inflammatory IL-1β, IL-6, IL-23α, and TNF-α cytokines from peritoneal macrophages, bone mononuclear cells, and colonic epithelial cells isolated from Acer3^-/-^ mice [105]. Overall, the bacterial species inhibit ceramidase activity to reduce the concentration of SPH in the host cells, which results in a reduced immune response. 

### 5.2. Role of Ceramidase Activity in Viral Infection 

Viruses have the potential to spread among individuals, resulting in epidemics that cause loss of human life and heavy burdens to healthcare systems [106]. The influenza, Ebola, and Zika epidemics are recent examples of the effects of broad viral infection and of the mechanisms by which viruses can be studied and controlled [106,107,108]. A recent mutation of the coronavirus, named SARS-CoV-2, has caused a pandemic of unprecedented magnitude. This virus has a lower mortality rate but is exponentially more contagious than the closely related SARS-CoV and MERS-CoV [109]. However, our knowledge of the potential role of host ceramidases in viral pathology remains elusive. It was reported that the overall inhibition of ceramidase activity in host peripheral blood lymphocytes using Ceranib 1 and Ceranib 2 significantly reduces the replication of the rhinovirus and measles virus, respectively [110,111]. Furthermore, the inhibition of ACDase activity in macrophages significantly increases the propagation of herpes simplex virus-1, which, in turn, elevates the mortality rate in Asah1^−/−^ mice [112]. 

Collectively, these published observations indicate that ceramidases may have an important antiviral effector role that should further studied.

## 6. Role of Ceramidases in Tissue Regeneration and Healing

Ceramidases are expressed in epithelial cells and fibroblasts and may be involved in their response through S1P [12,103,113]. However, only a limited number of studies have demonstrated the effects of these enzymes in tissue regeneration and healing. 

### 6.1. Acid Ceramidase

ACDase activity contributes to physiological processes involving collagen turnover. ASAH1 is associated with familial keloid healing and is overexpressed in keloid scar tissue and hypertrophic scars caused by excessive collagen deposition during epidermal healing [114]. In the liver, hepatic stellate cells (HSC) are activated during normal wound healing but can, after multiple activations, cause hepatic fibrosis. However, the inhibition of ACDase by tricyclic antidepressants leads to ceramide accumulation, which inactivates HSCs and prevents hepatic fibrosis [115]. In vivo studies have also demonstrated a positive effect of ACDase in chondrocyte differentiation. In cartilage replacement therapy, pre-treatment with ACDase induces chondrocyte proliferation, the production of glycosaminoglycan, the expression of *COL2*, the adhesion of chondrocytes to a scaffold, a reduced resorption after implantation, and an improved differentiation to cartilage [116]. Furthermore, a variation of FRBRL characterized by peripheral osteolysis not associated with MMP-2 and MMP-14 was found, suggesting the involvement of ASAH1 in bone remodeling [117].

### 6.2. Neutral Ceramidase

Various studies have focused on the use of exosomes for tissue repair and regeneration [118,119]. The results of a recent study indicated that hepatocyte exosomes show significant NCDase activity and promote hepatocyte proliferation in vitro and liver regeneration in vivo [118]. This suggests a role of NCDase in tissue regeneration. The ceramidase has also been identified as an antagonist of cell necrosis caused by 2DG/AA-dependent ceramide accumulation and mitochondrial damage [34]. Furthermore, NCDase increases autophagy and protects cells from ER stress-mediated cell death [34]. 

### 6.3. Alkaline Ceramidase

ACER1 inhibition leads to abnormal hair, alopecia, hyperproliferation, inflammation, an abnormal differentiation of the epidermis, sebaceous gland abnormalities, and infundibulum expansion, as well as an increased trans-epidermal water loss and hypermetabolism with an associated reduction in fat content during aging [12]. Its inhibition gradually depletes the number of hair follicle stems and causes alopecia through decreased hair follicle activity [14]. The specific mechanisms through which these effects of ALKCDase occur are still not detailed in the literature. However, its expression has been associated with keratinocyte growth arrest and differentiation [13]. Altogether, these data suggest that ACDase is involved in collagen matrix metabolism, whereas NCDase and ALKCDase appear to affect tissue regeneration and healing through their anti-apoptotic effects. 

## 7. Conclusions

**Figure 4:**
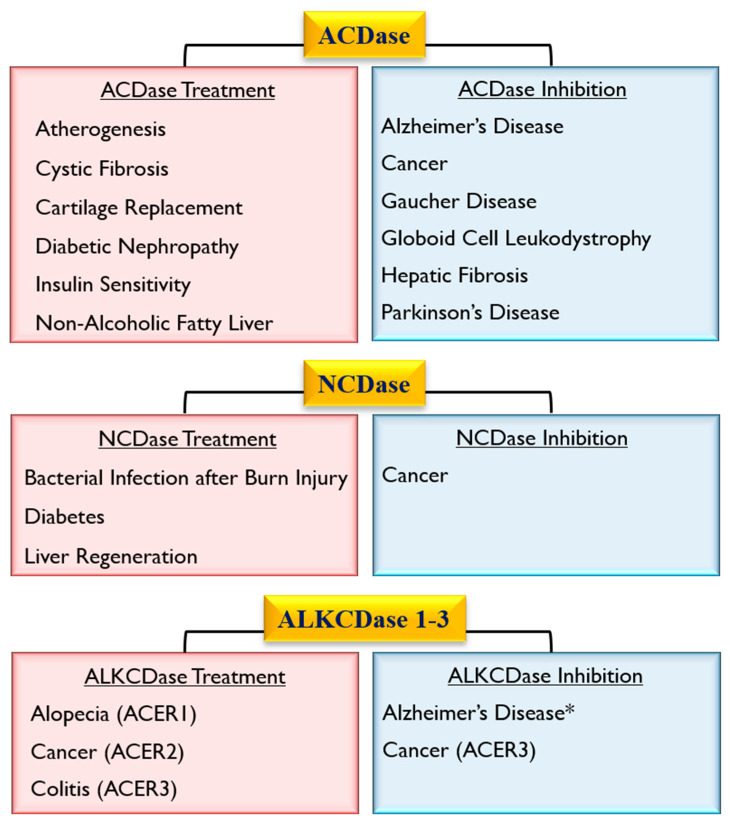
Potential therapeutic targeting of acid (ACDase), neutral (NCDase), and alkaline (ALKCDase) ceramidases. *Unspecified class of ALKCDase [80].

Ceramidases (acid, neutral, alkaline) are key enzymes that maintain the intracellular homeostasis of ceramide/SPH and are critical regulators of signals that tilt the balance between cell survival and death. Various studies have demonstrated the involvement and potential therapeutic role of these enzymes in a diverse set of common human diseases, including bacterial-induced infectious diseases, neurodegenerative diseases, cancer, diabetes, and others (Figure 4). Therefore, the clinical applicability of studies examining the versatility of the effects of ceramidases in health and disease deserves further examination.
